# Neatness in Dress at Home

**Published:** 1890-12

**Authors:** 


### NEATNESS IN DRESS AT HOME.

The importance of neat and tasteful house dressing cannot be overestimated. The matron who appears before the members of the family in a shabby soiled wrapper, and makes the excuse, if, indeed, she takes the trouble to make one at all, that "it is so much more comfortable," has little idea of the possible consequence of such a course. Could she but realize that her dress is an evil example to her daughters, and one productive of consequences that will reach far beyond her own span of life; that her husband and sons cannot fail to draw comparisons between her dress and that of the ladies they meet in other homes, and that these comparisons cannot fail to decrease their respect for her, she might be induced to give more attention to her personal appearance. Not even the burden of care and constant employment can furnish a sufficient excuse for careless personal habits, for few things are more important to the well being of a family. There is an old saying to the effect that an untidy mother has disobedient children; and, while neither parents nor children may realize the why or wherefore of it, yet there is always a lack of respect and an indifference to the authority of a mother who takes no pride in her personal appearance.

And it is not the mother alone upon whose shoulders rests the burden of responsibility for home neatness and order in dress; the father has his duties to look after as well, and should never fail to insist upon the younger members of the family presenting themselves with well kept hands, clean faces, neatly brushed hair, and orderly dress, at least at every meal where the family assembles.—*Christian Leader.*

EFFECTS OF QUININE.—Dr. Barton, of Mississippi, in the Memphis *Journal of the Medical Science,* last March, charged that malarial hematuria, a disease prevalent in the low river country of the South, was really nothing but cinchonism, due to the "absurd and criminal quantity" of quinine used. He stated that he is fresh from the teachings of late authorities in medicine, but has had to unlearn much about the use of quinine.

